# Regional and National Trends in Consumption of Antimicrobials in Pakistan; Pre and Post-COVID (2019–2021)

**DOI:** 10.1093/cid/ciad647

**Published:** 2023-12-20

**Authors:** Tauqeer Mustafa, Muhammad Rehan Khan Niazi, Zahra Lakdawala, Shaper Mirza

**Affiliations:** Fleming Fund Country Grant, DAI, Islamabad, Pakistan; Department of Pharmacy, Shaukat Khanum Hospital and Research Center, Lahore, Pakistan; Numerical Yield and Site Assessment Group, Fraunhofer Institute for Wind Energy Systems, Oldenburg, Germany; Department of Life Sciences, SBASSE-LUMS, Lahore, Pakistan

**Keywords:** antimicrobials, antibiotics consumption, trend analysis, COVID-19, Pakistan

## Abstract

**Background:**

Efforts to combat antimicrobial resistance, a growing public health problem in Pakistan, have been hampered by the lack of high-quality national and provincial-level antimicrobial consumption data. The singular objective of this retrospective study was to measure antimicrobial consumption over 3 years between 2019 and 2021.

**Methods:**

The study was designed to estimate antimicrobial consumption at National and Regional levels. Antimicrobial consumption data was collected by IQVIA covering 110 districts of Pakistan in which 88% of sales are census (accurate sales collected directly from distributors), whereas 12% of sales (sales of 300 pharmacies) are projected on the national level. To determine the usage for 3 consecutive years, the consumption of antibiotics was calculated as defined daily doses (DDD) of antibiotics per 1000 inhabitants per day (DID).

**Results:**

The results of our study demonstrated a steep increase in the consumption of antimicrobials from 2019 to 2021. An increase in consumption of most classes of antibiotics was observed both nationally and Regionally. Quinolones, penicillins (co-amoxiclav), macrolides, and third-generation cephalosporins remained the most frequently used antibiotics nationally. A 40% increase in intravenous use of antimicrobials was observed between 2019 and 2021 at the national level. Moxifloxacin, Levofloxacin, Ciprofloxacin, and linezolid were the most commonly used intravenous antibiotics. Region 7 (Peshawar) demonstrated the highest consumption, followed by Region 1 (Karachi) and Region 6 (Faisalabad). Among the most commonly used antibiotics, the use of third-generation cephalosporin (cefixime), quinolones, penicillins (amoxicillin + clavulanic acid), and macrolides (azithromycin) was most noticeable in all regions, particularly in those with the higher consumption of antibiotics.

**Conclusions:**

Although the increase in consumption of all antibiotics is concerning, the steep increase in the use of watch and reserve category antibiotics during the study period calls for immediate actions to limit and regulate their usage.

An estimated 1.27 million individuals including 214 000 newborns lose their lives to resistant infections every year gobally [1]. Although the Center for Disease Control and Prevention (CDC) reported 2.8 million infections with 35 000 deaths per year from resistant infections in The United States, the European Union (EU)/European Economic Area witnessed 670 000 infections and 33 000 deaths [2, 3]. Overall, global numbers of resistant infections are expected to reach 10 million by 2050, costing the global economy approximately 100 trillion through loss of productivity. The Organisation of Economic Co-operation and Development (OECD)/World Bank Group projected that Europe, North America, and Australia will have 2.4 million deaths each year due to antimicrobial resistance (AMR) [4–8].

Pakistan is the fifth most populous country in the world, going to become fourth most populous by 2050. World Health Organization ranks Pakistan fifth in the burden of multidrug-resistant tuberculosis. Additionally, the country has experienced 2 outbreaks of multidrug-resistant *Salmonella* infections (100% resistant to fluoroquinolones) and is still struggling with antimicrobial-resistant pneumococcal infections, which kill 20 000 children under the age of 5 years [9–12]. The burden of hospital acquired infections (HAIs) or nosocomial infections in Pakistan is not properly documented, yet the worrisome rise of multidrug-resistant organisms (MDROs) (*Acinetobacter baumannii*, *Pseudomonas aeruginosa*, *Klebsiella* species, *Escherichia coli* and methicillin-resistant *Staphylococcus aureus* [MRSA], etc.) suggests that the risk of acquiring infection may be as high as 60% for some hospitalized patients with an attributable mortality rate of 12%–60% in some populations [13, 14].

Pakistan is the third largest consumer of antibiotics in low- to middle-income countries. An estimated 50% of these antibiotics are inappropriately prescribed [15–17]. Drivers of the emergence of antimicrobial resistance in Pakistan are embedded in both social awareness and antimicrobial usage. Social contributors to the crisis include poverty, false beliefs and rituals, resulting in non-compliance with the use of antibiotics.

Inappropriate antibiotic usage was also witnessed during the coronavirus disease 2019 (COVID-19) pandemic in Pakistan. Antibiotics were administered to hospitalized patients to prevent secondary bacterial infections [18–20]. Empirical treatment of suspected bacterial infections in COVID-19 patients was also observed in several tertiary care facilities. Studies conducted in tertiary care facilities in major cities of Pakistan demonstrated an increase in usage of azithromycin (11.5-17 DDD/100 occupied bed-days), ciprofloxacin (3.5–3.8 DDD/100 occupied bed-days), ceftriaxone (20–25 DDD/100 occupied bed-days), and piperacillin-tazobactam (0.3–1 DDD/100 occupied bed-days) [21–23].

These findings suggest that the current antimicrobial stewardship (AMS) practices are either ineffective or are not being implemented appropriately to curb the indiscriminate usage of antibiotics. The situation therefore mandates routine investigation into antimicrobial usage in tertiary care facilities and pharmacies, to improve patient safety and clinical outcomes.

To understand usage trends in Pakistan, we performed an analysis of antibiotic usage data, collected between 2019 and 2021.

## METHODS

### Study Design

A retrospective study was performed to measure national consumption of antibiotics between the years 2019 and 2021. For this study data were collected by IQVIA through a retail sales audit in which 88% of sales are census (accurate sales collected directly from distributors), whereas 12% of sales (sales of 300 pharmacies) are projected on the national level. Nearly all big companies' sales data are collected directly from distributors and validated. IQVIA does also validate the sales data of the companies whose data are projected from a panel of pharmacies and the overall accuracy and precision of data is ∼95%. Data collected represent usage for 3 full calendar years (365 days/year). Data from all sources were combined to calculate national averages. Regional consumption was estimated by dividing the country into 8 Regions as follows: Region 1, Karachi; Region 2, Sindh (excluding Karachi); Region 3, Multan; Region 4. Lahore; Region 5, Faisalabad; Region 6, Rawalpindi; Region 7, Peshawar; and Region 8, Baluchistan.

### Statistical Analysis for Antibiotic Consumption

Data were analyzed for measuring (i) antibiotic consumption and for (ii) identifying trends of consumption between 2019 and 2021. For consumption, defined daily doses (DDD) were calculated from the consumption data representing antibiotic consumption both nationally and regionally. The DDD per 1000 inhabitants per day was calculated for all antibiotics for 3 consecutive years (2019, 2020, 2021) following the ATC and DDD methodology approved by the World Health Organization (WHO). For antibiotics where the ATC codes were not defined by WHO (eg, Co-trimoxazole), routine daily dosages were used. All data analysis was done using the Python statistical package. For calculating DDD, the following formula was used.


DDD=Grams/ATCCodes

For calculating national DIDs, the population size used was 198 million (2019), 201 million (2020), and 205 million (2022). For calculating Regional DIDs we used the population of each region as a denominator.

Defined Daily Doses per 1000 Inhabitants/day (DIDs) were calculated using the following formula:


$$\mathrm{DDD}\;\mathrm{per}\;1000\;\mathrm{inhabitants}\;\mathrm{per}\;\mathrm{day}=\frac{\mathrm{DDD}\times 1000}{\mathrm{Population}\times 365}\;$$

Cumulative DIDs for 3 study years are presented as line graphs to demonstrate differences between overall antibiotic consumption during the 3-year study period. Percentages were calculated to demonstrate the top 5, most commonly used antibiotics nationally during the 3-year study period. Percentages were also used to present DIDs of the most commonly used antibiotics regionally. Geographic information system (GIS) maps were constructed to identify areas of highest consumption within regions. One-way analysis of variance (ANOVA) was used to compare the DID between the study period and the comparison was considered significant if *P* < .05.

## RESULTS

### Cumulative Consumption of Antibiotics-National Estimates

**Figure 1.**
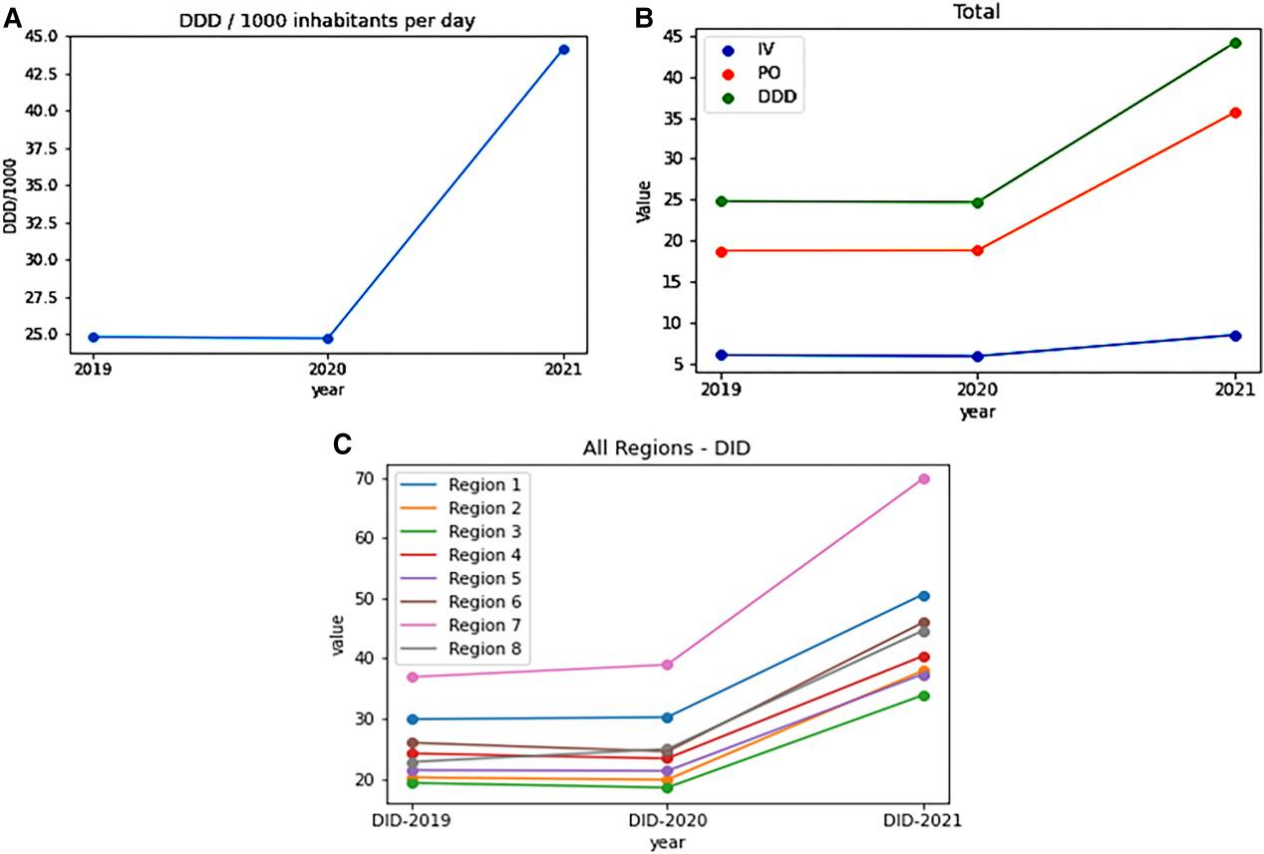
*A*, DID values presented by year. Increase in consumption was observed between 2020 and 2021. *B*, National DID values for cumulative per-oral and intravenous consumption. Highest use was observed through per-oral route. *C*, DID values by Region. Region 7 had the highest DID value (70) followed by Region 1 (50) and Region 6 (45). Abbreviations: DDD, defined daily dose; DID, daily dose per 1000 inhabitants per day.

Antibiotic consumption was calculated for all antibiotics used during the study period of 3 years (2019–2021). Overall national consumption increased from 24.8 DID in 2019 to 44.1DIDs in 2021 (Figure 1*A*), which is also reflected in the increase in both per-oral and intravenous administration (Figure 1*B*). Regional consumption also followed the same trend, demonstrating increasing usage of antibiotics between 2020 and 2021 (Figure 1*C*). For national estimates, cumulative DID represents the overall consumption of all antibiotics and showed an increase of 20 DID between years 2020 and 2021. When stratified by route of administration, an increase from 5.9 DID to 8.5 DID was observed between 2020 and 2021 in intravenous administration. The Increase in oral consumption (18 DID in 2020, 36 DID in 2021) was 2-fold and was significantly greater as compared to intravenous consumption Table 1 (*P*-values .01).

**Table 1.** Comparison of DID of Most Commonly Used Antibiotics Between 2019 and 2021

Antibiotics	DID 2019	DID 2020	DID 2021	Net Increase in DID (%)	*P* Value
Total	24.8	24.7	44.1	19.3 (79%)	<.001
Per oral	18.8	18.8	35.6	16.8 (89.6)	<.001
Intravenous	6.0	5.9	8.5	2.5 (40)	.050
Macrolides	2.6	3.4	6.1	3.5 (135)	.067
Third gen cephalosporin	2.5	2.9	7.2	4.7 (182)	.057
Carbapenems	0.01	0.01	0.01	NA	NA
Amoxicillin + Co-amoxiclav	3.8	3.8	8.7	4.9 (118)	.039
Aminoglycosides	0.1	0.1	0.1	…	.014
Quinolones	9.3	9.0	12.6	3.3 (35)	.009
Anti-MRSA^a^	0.7	0.6	1.2	0.5 (80)	.985
Tetracycline	2.3	2.2	2.3	…	.161
Penicillin	4.3	4.2	9.1	4.8 (110)	.028
PEP-TAZ	0.01	0.01	0.02	0.01 (183)	.956
First and second gen cephalosporin	1.0	1.0	2.2	1.2 (115)	.844
Sulfamethoxazole + trimethoprim	1.4	1.4	2.7	1.3 (90)	.123

Abbreviations: DID, daily dose per 1000 inhabitants per day; MRSA, methicillin-resistant *Staphylococcus aureus*.

^a^Anti-MRSA-vancomycin, daptomycin, Sulfamethoxazole + trimethorpim, clindamycin and tigecycline.

Evaluation of national averages of commonly used classes of antibiotics from 2019 to 2021 demonstrated a 2–3 times increase in usage of penicillin (co-amoxiclav), third generation cephalosporin (cefixime), macrolides (azithromycin), and carbapenems. Although we did observe an increase in use of carbapenems in 2021, the scale was not significant owing to the overall low proportion of this class to the total antimicrobial consumption.

### Cumulative Consumption of Most Frequently Used Antibiotics Nationally

Data presented in Table 1 demonstrate national trends in the utilization of antimicrobials during a 3-year study period. The most frequently used antibiotics over the 3-year study period, were cefixime, co-amoxiclav (amoxicillin + clavulanic acid), ciprofloxacin, moxifloxacin, levofloxacin, azithromycin, doxycycline, and linezolid, respectively. Consumption of ciprofloxacin was highest between 2019 and 2021, followed by levofloxacin, moxifloxacin, and amoxicillin + clavulanic acid. In comparison to 2019 and 2020, usage of cefixime increased in 2021 With an overall increase of 1.5 DID, macrolide consumption was most noticeable between 2019 and 2020, which increased to 2.7 DID in 2021. Finally, a 4 DID increase in third-generation cephalosporin was observed from 2020 to 2021.

### National Consumption of Antibiotics by Route of Administration-per-oral Versus Intravenous

Significantly high oral consumption of amoxicillin + clavulanic acid was observed between 2019 and 2020, followed by doxycycline and ciprofloxacin. In the year 2020, azithromycin remained the second most consumed antibiotic, with 3 times higher consumption between 2020 and 2021 as compared to 2019 and 2020. A 3-fold increase in consumption of cefixime was also observed from 2020 to 2021.

Interestingly, oral consumption of doxycycline remained static throughout the 3-year study period.

Relative to oral consumption, intravenous consumption of antibiotics remained low nevertheless, a marginal increase in administration through intravenous routes was observed from 2019 to 2021. Higher consumption of quinolones (moxifloxacin, levofloxacin, and ciprofloxacin) was observed from 2019 to 2021 (DID 9.4 in 2019, DID 8.7 in 2020, and DID 12.6 in 2021; Table 1). Although intravenous consumption of levofloxacin and ciprofloxacin remained consistent over the 3 years, a 2-fold increase in consumption of moxifloxacin was observed from 2020 to 2021 (DID 2 in 2020, and DID 4 in 2021). In 2021 consumption of linezolid increased significantly making it the fourth most consumed antibiotic (DID 0.5 in 2020, DID 1.04 in 2021).

### Three-year Trends of Regional Antibiotic Consumption

**Figure 2.**
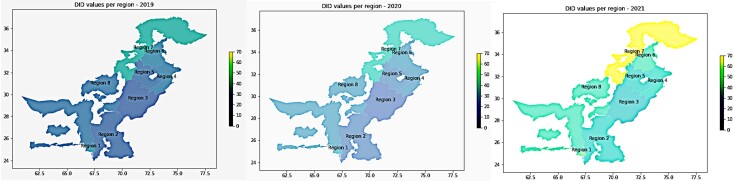
GIS map of country demonstrating Region-wise DID values. GIS maps representing areas of highest consumption (2019–2021). Abbreviations: DID, daily dose per 1000 inhabitants per day; GIS, geographic information system.

Regional consumption trends remained consistent with national trends in that the highest consumption of antibiotics was observed between 2020 and 2021. Stratification by regions indicated that the consumption differs greatly by region (Figure 2 and Table 2). Region 7 (Peshawar) showed high consumption of antibiotics for all 3 years (DID per year was 35, 40, and 70 for 2019, 2020, and 2021, respectively). Penicillin, sulphamethoxazole + trimethoprim, and cephalosporins were the most frequently used antibiotics in Region 7, followed by cefixime and amoxicillin-clavulanic acid. After Region 7, Region 1 (Karachi), and Region 6 (Rawalpindi) showed high consumption. Unlike Region 7, where penicillin was the most frequently used, Region 1 and Region 6 showed increased usage of quinolones, linezolid, and piperacillin-tazobactam between 2020 and 2021.

**Table 2.** Regional Consumption Trends for Three Years (2019–2021)

	2019	2020	2021
Region 1, Karachi (net increase in 3 y DID + %)			
Cumulative (21.3 DID, 71%)	Amoxicillin + clavulanic acid (12.1%) Doxycycline (9.3%) Cefixime (10%)	Amoxicillin + clavulanic acid (10.2%) Azithromycin (10.3%) Cefixime (9.5%)	Amoxicillin + clavulanic acid (13%) Azithromycin (9.7) Cefixime (15.7%)
Per oral (18.25 DID, 83%)	Amoxicillin + clavulanic acid (16%) Cefixime (13%) Doxycycline (11.9%)	Amoxicillin + clavulanic acid (13.6%) cefixime (12%) Azithromycin (13.8%)	Amoxicillin + clavulanic acid (16.3%) Cefixime (20.5%) Azithromycin (12.2%)
Intravenous (2.45, DID, 30%)	Linezolid (21%) Ciprofloxicin (22%) Moxifloxacin (29.5%)	Linezolid (16%) Ciprofloxacin (21.9%) Moxifloxacin (34.2%)	Linezolid (34%) Ciprofloxacin (17.9%) Moxifloxacin (30.6%)
Region 2, Sindh			
Cumulative (17.6, DID, 83%)	Ciprofloxacin (23.1%) Cefixime (15.4%) Moxifloxacin (15.4%) Levofloxacin (15.4%)	Ciprofloxacin (25%) Cefixime (16.7%) Moxifloxacin (16.7%) Levofloxacin (16.7%)	Ciprofloxacin (17.9%) Cefixime (25%) Azithromycin (10.7%) Amoxicillin-Clavulanic acid (10.7%)
Per oral (16.25 DID, 105%)	Cefixime (25%) Azithromycin (12.5%) Levofloxacin (12.5%) Amoxicillin-Clavulanic acid (12.5%)	Cefixime (28.6%) Azithromycin (14.3%) Levofloxacin (14.3%) Amoxicillin-Clavulanic acid (14.3%)	Cefixime (30.4%) Azithromycin (13%) Ciprofloxacin (13%) Amoxicillin-Clavulanic acid(13%)
Intravenous (1.39, DID, 28%)	Moxifloxacin (37.2%) Ciprofloxacin (28.1%) Levofloxacin (13%)	Moxifloxacin (36.2%) Ciprofloxacin (27.1%) Levofloxacin (14.5%)	Moxifloxacin (39.2%) Ciprofloxacin (22.1%) Levofloxacin (15.9%)
Region 3, Multan			
Cumulative (14.55, DID, 75%)	Ciprofloxacin (27.3%) Levofloxacin (18.2%) Moxifloxacin (18.2%)	Ciprofloxacin (27%) Moxifloxacin (18.2%) Doxycycline (9.1%) Amoxicillin-Clavulanic acid (9.1%)	Ciprofloxacin (17.4%) Cefixime (17.4%) Moxifloxacin (13%) Amoxicillin-clavulanic acid (13%)
Per oral (12.5, DID, 91%)	Ciprofloxacin (18%) Levofloxacin (16.7%) Doxycycline (16.7%) Amoxicillin-Clavulanic Acid (16.7%)	Ciprofloxacin (15%) Levofloxacin (14.3%) Doxycycline (14.3%) Amoxicillin-Clavulanic Acid (14.3%)	Cefixime (22%) Amoxicillin-clavulanic acid (19%) Ciprofloxacin (16.7%)
Intravenous (2, DID, 37%)	Moxifloxacin (35.4%) Ciprofloxacin (27.2%) Levofloxacin (14.3%)	Moxifloxacin (33.4%) Ciprofloxacin (27.9%) Levofloxacin (14%)	Moxifloxacin (33.4%) Ciprofloxacin (27.9%) Levofloxacin (14%)
Region 4, Lahore			
Cumulative (16.06, DID, 66%)	Moxifloxacin (15.9%) Ciprofloxacin (13.9%) Levofloxacin (10.2%) Amoxicillin-Clavulanic Acid (8.6%)	Moxifloxacin (15.7%) Ciprofloxacin (12.7%) Levofloxacin (9.9%) Amoxicillin-Clavulanic Acid (8.5%)	Moxifloxacin (15.7%) Cefixime (14.6%) Amoxicillin-clavulanic acid (11.5%)
Per oral (13.25, DID, 75%)	Amoxicillin-clavulanic acid (12.5%) Ciprofloxacin (11.8%) Cefixime (11%)	Azithromycin (12%) Amoxicillin-clavulanic acid (11.2%) Cefixime (11%)	Cefixime (19.4%) Amoxicillin-clavulanic acid (15.2%) Azithromycin (11.3%)
Intravenous (2.62, DID, 37%)	Moxifloxacin (48.4%) Ciprofloxacin (18.1%) Linezolid (10.3%)	Moxifloxacin (51.2%) Ciprofloxacin (17.03%) Linezolid (10.8%)	Moxifloxacin (61%) Ciprofloxacin (12.8%) Linezolid (11.2%)
Region 5, Faisalabad			
Cumulative (15.9, DID, 74%)	Moxifloxacin (15.9%) Ciprofloxacin (15.4%) Levofloxacin (10.3%)	Moxifloxacin (16.4%) Ciprofloxacin (14.3%) Levofloxacin (10.8%)	Moxifloxacin (16.2%) Cefixime (13%) Amoxicillin-clavulanic acid (9.9%)
Per oral (12, DID, 80%)	Ciprofloxacin (11.9%) Doxycycline (11.6%) Levofloxacin (11.4%)	Ciprofloxacin (12%) Doxycycline (11.6%) Amoxicillin-clavulanic acid (10.4%)	Cefixime (17.8%) Amoxicillin-clavulanic acid (13.5%) Ciprofloxacin (9.8%)
Intravenous (3.6, DID, 57%)	Moxifloxacin (47.3%) Ciprofloxacin (22%) Levofloxacin (10.1%)	Moxifloxacin (48.1%) Ciprofloxacin (18.1%) Linezolid (11.9%)	Moxifloxacin (54.5%) Ciprofloxacin (13.4%) Linezolid (11.8%)
Region 6, Rawalpindi			
Cumulative (19.9, DID, 76%)	Moxifloxacin (12.5%) Ciprofloxacin (11.9%) Levofloxacin (11.7%)	Moxifloxacin (11.9%) Levofloxacin (11.6%) Amoxicillin + clavulanic acid (11.2%)	Amoxicillin + clavulanic acid (14.5%) Cefixime (14.2%) Moxifloxacin (11.2%)
Per oral (17.2, DID, 88%)	Amoxicillin + clavulanic acid (14%) Doxycycline (12.2%) Cefixime (10.9%) Ciprofloxacin (10.5%)	Amoxicillin + clavulanic acid (14.5%) Azithromycin (14.1%) Doxycycline (12.2%)	Amoxicillin + clavulanic acid (17.5% Cefixime (16.3%) Azithromycin (11.6%)
Intravenous (2.73, DID, 42%)	Moxifloxacin (45%) Levofloxacin (17.6%) Ciprofloxacin (17.1%)	Moxifloxacin (43%) Levofloxacin (20%) Ciprofloxacin (16.1%)	Moxifloxacin (51%) Levofloxacin (18%) Linezolid (10%)
Region 7, Peshawar			
Cumulative (33, DID, 89%)	Amoxicillin + clavulanic acid (13.3%) Ciprofloxacin (13.2%) Doxycycline (12.3%)	Ciprofloxacin (14.8%) Amoxicillin + clavulanic acid (14.8%) Doxycycline (11.9%)	Amoxicillin + clavulanic acid (17.1%) Cefixime (12.4%) Ciprofloxacin (10.6%) Amoxicillin (10.5%)
Per oral (30, DID, 94%)	Amoxicillin + clavulanic acid (15.4%) Doxycycline (14.2%) Amoxicillin (11.6%)	Amoxicillin + clavulanic acid (14.8%) Doxycycline (14.7%) Amoxicillin (10.7%)	Amoxicillin + clavulanic acid (19.4%) Cefixime (14.1%) Amoxicillin (11.9%)
Intravenous (2.89, DID, 55%)	Ciprofloxacin (34.2%) Moxifloxacin (26.9%) Levofloxacin (11.5%)	Moxifloxacin (35.9%) Ciprofloxacin (28.1%) Levofloxacin (11%)	Moxifloxacin (43.1%) Ciprofloxacin (22%) Levofloxacin (9%)
Region 8,Baluchistan			
Cumulative (21.7, DID, 95%)	Ciprofloxacin (17.6%) Levofloxacin (10.7%) Amoxicillin + clavulanic acid (10.0%)	Ciprofloxacin (16%) Levofloxacin (10.7%) Azithromycin (8.7%) Amoxicillin + clavulanic acid (8.3%)	Cefixime (14.9%) Ciprofloxacin (14.2%) Amoxicillin + clavulanic acid (11.3%)
Per oral (19.58, DID, 107%)	Ciprofloxacin (12.4%) Amoxicillin + Clavulanic acid (12.1%) Cefixime (10.5%)	Ciprofloxacin (12.1%) Azithromycin (11%) Amoxicillin + Clavulanic acid (10.5%) Cefixime (9.3%)	Cefixime (17.2%) Amoxicillin + Clavulanic acid (13.1%) Ciprofloxacin (10.9%)
Intravenous (1.82, DID, 42%)	Ciprofloxacin (40%) Levofloxacin (21%) Moxifloxacin (19.6%)	Ciprofloxacin (37%) Moxifloxacin (24.1%) Levofloxacin (19.8%)	Ciprofloxacin (33.9%) Moxifloxacin (31.3%) Levofloxacin (18.1%)

Abbreviation: DID, daily dose per 1000 inhabitants per day.

### Antibiotic Consumption in Region 1 (Karachi)

Results of regional consumption are presented in Table 2. Overall consumption of antibiotics was higher in Region 1 and exceeded national consumption. A net increase of 21% in DID was observed in the total consumption of antimicrobials in Region 1 from 2019 to 2021. A review of cumulative consumption demonstrated that amoxicillin + clavulanic acid was the most frequently consumed antibiotic in 2019–2020 followed by cefixime, azithromycin, and doxycycline. A total of 83% (net increase of 18.3 DID) increase in oral consumption, and a 30% increase in the consumption of intravenous antimicrobials was observed between 2020 and 2021. Additionally, per-oral consumption trends were comparable to cumulative consumption where cefixime showed the highest overall consumption from 2020 to 2021. The year-wise analysis demonstrated that between 2019 and 2020 moxifloxacin was the most commonly used IV antimicrobial followed by ciprofloxacin and linezolid. In 2021, linezolid became the most commonly used antibiotic with approximately 34% of all intravenously used antibiotics followed by moxifloxacin and ciprofloxacin. Another important observation was the 2 to 3fold increase in the consumption of azithromycin from 2020 to 2021, in comparison to pre-COVID19 years (2019–2020). Similarly, penicillins, amoxicillin + clavulanic acid, cefixime, and linezolid showed significantly higher usage in the year 2021 as compared to the years 2019 or 2020.

### Antibiotic Consumption in Region 2 (Sindh, Excluding Karachi)

An increase in both per-oral and intravenous consumption was noticed between 2020 and 2021. A significant increase in the usage of penicillins—cephalosporin, macrolides, linezolid, and carbapenems—was observed from 2019 to 2021. Ciprofloxacin remained the most consumed antibiotic followed by cefixime, moxifloxacin, and levofloxacin until the year 2020 after which it was replaced by cefixime, azithromycin, and amoxicillin + clavulanic acid in the year 2021. Cefixime alone constituted one-fourth (25%) of the cumulative consumption of antibiotics in Sindh. Intravenous consumption of moxifloxacin remained high which was 37% of all intravenously consumed antibiotics in the year 2019, the usage further increased in the year 2020 and reached a maximum of 39.2% in year 2021.

### Antibiotic Consumption in Region 3 (Multan)

Consumption of antibiotics in Region 3 remained comparable for the years 2019–2020 but changed drastically for the year 2021. Overall, a sharp increase (5.1 DID to 7.5 DID) was observed in the year 2021. Quinolones remained the most frequently used antibiotic via intravenous route. The most consumed quinolone was moxifloxacin, constituting one-third (33%) of all intravenously consumed antibiotics, followed by ciprofloxacin and levofloxacin. Ciprofloxacin was the drug of choice for per-oral use followed by amoxicillin + clavulanic acid and doxycycline in 2019; however, between 2020 and 2021, azithromycin consumption spiked. Additionally, a more than 2-fold increase in penicillins—cephalosporin, carbapenems, and linezolid—was observed between years 2020 and 2021 as compared to the year 2019.

### Antibiotic Consumption in Region 4 (Lahore)

An estimated 66% increase in cumulative consumption of antibiotics was observed in Region 4 in the year 2021 as compared to the year 2019. A 78% increase in oral and 35% increase in intravenous consumption of antibiotics was observed from 2019 to 2021. Cumulative consumption indicated a significant increase in consumption of macrolides, cephalosporins, penicillin, and quinolones. Among quinolones, moxifloxacin and ciprofloxacin were the most commonly used antibiotic in Region 4 throughout the study period. The consumption pattern from 2020 to 2021 showed a shift toward consumption of cefixime and amoxicillin + clavulanic acid, replacing ciprofloxacin. Moxifloxacin remained the antibiotic of choice for intravenous administration and constituted 50%–60% of all intravenously administered antimicrobials. Ciprofloxacin and linezolid were the second and third most consumed intravenous antibiotics.

### Antibiotic Consumption in Region 5 (Faisalabad)

Except for aminoglycosides and tetracyclines, consumption of all other antibiotics increased over the 3 years from 2019 to 2021. In particular, a 2-fold increase was observed in the consumption of cephalosporin, penicillin, macrolides, and linezolid. The most frequently used antibiotics in Region 5 were moxifloxacin, ciprofloxacin, amoxicillin + clavulanic acid, cefixime, and levofloxacin. A net increase of 15.4 DID (74%) in consumption of moxifloxacin was observed in the year 2021, where it remained the second most consumed antibiotic. Among oral antibiotics, ciprofloxacin was the most commonly consumed antibiotic from 2019 to 2020; however, it was replaced by cefixime and amoxicillin + clavulanic acid in 2021. Moxifloxacin, followed by ciprofloxacin and linezolid, remained the drugs of choice for intravenous use.

### Antibiotic Consumption in Region 6 (Rawalpindi)

In comparison to other regions, Region 6 demonstrated a higher antimicrobial consumption after Region 7. An increase in consumption of penicillin, macrolides, quinolones, carbapenem, co-trimoxazole, and linezolid was observed from 2019 to 2021. A net increase of 19.9 DID (76%) was observed over the 3 years (2019–2021), with the most significant increase between 2020 and 2021. An increase of 88% was observed in the consumption of oral antibiotics, whereas intravenous consumption increased by 42% from 2019 to 2021. Quinolones remained the drug of choice during 2019–2020, whereas in 2021 amoxicillin + clavulanic acid together with cefixime and moxifloxacin showed higher consumption. Orally, consumption of amoxicillin + clavulanic, azithromycin, doxycycline, cefixime, and ciprofloxacin and intravenous administration of quinolones were frequently reported. In 2021, linezolid replaced ciprofloxacin to become the third most commonly used antibiotic.

### Antibiotic Consumption Region 7 (Peshawar)

The cumulative increase in antimicrobial consumption in Region 7 was 32.8 DID (90%), from 2019 to 2021, which is the highest of all 8 regions analyzed. Analysis by route of administration indicated a 94% increase in consumption of oral antibiotics where the most frequently used antibiotics between 2019 and 2020 were amoxicillin + clavulanic acid, doxycycline, amoxicillin, and cefixime in 2021. A 55% increase in consumption of intravenous antibiotics was observed, whereas quinolones remained the class of choice. Among quinolones, moxifloxacin was used most frequently followed by ciprofloxacin and levofloxacin.

### Antibiotic Consumption Region 8 (Baluchistan)

The most important observation for Region 8 was the overall highest increase in consumption of antibiotics (95%) from 2019 to 2021. The increase in consumption of oral antibiotics was most noticeable with a net increase of 19.85 DID (107%). Ciprofloxacin remained the most commonly used per-oral antibiotic during 2019–2020 followed by amoxicillin + clavulanic acid. Cefixime consumption increased in 2021 replacing ciprofloxacin. Among quinolones, the most commonly administered quinolone via intravenous route was ciprofloxacin, followed by moxifloxacin and levofloxacin. Overall consumption of penicillins (including anti-pseudomonal penicillins)—co-trimoxazole, cephalosporins, macrolides, carbapenems, and linezolid—increased significantly.

## DISCUSSION

Pakistan has one of the highest burdens of infectious diseases in the world [13, 14, 24]. To make matters worse, growing economic and financial fragility in the aftermath of the COVID-19 pandemic, combined with rapidly changing climate and natural disasters, put Pakistan at high risk of a national health crisis brought on by infectious diseases [25]. Efforts are being made both globally and locally to lower the burden of resistance by raising awareness regarding the non-judicious use of antimicrobials, developing a national action plan for monitoring, and managing resistance, improving infection prevention and control programs and estimating antimicrobial usage [26–28].

This study and those conducted earlier have demonstrated a significantly high consumption of WHO **access** (amoxicillin, amoxicillin + clavulanic acid, sufamethoxazole + trimethoprim and doxycycline), **watch** (ciprofloxacin, moxifloxacin, azithromycin, and cefixime), and **reserve** (linezolid, ceftazidime-avibactam and meropenem) groups of antibiotics [29, 30].

A review of antimicrobial usage at the national level demonstrated a steep increase in usage in the year 2021 as compared to the years 2020 or 2019, which might be a result of panic medication against the COVID threat. Between 2019 and 2021, usage of per-oral antimicrobials increased up to 3 times. The most commonly consumed antimicrobials between years 2019 and 2021, included quinolones, penicillins, macrolides, third-generation cephalosporins and tetracycline. Although consumption of third-generation cephalosporin as first line of therapy is inappropriate, however, the decision to use those was not the result of the unavailability or shortage of the first line of antibiotics; rather it was the choice of the prescriber. A comparable trend has been observed in several other countries of the world such as China, India, Scandinavia, and other Southeast Asian countries [31–34]. An interesting observation that emerged from our analysis was that among the most consumed antibiotics, consumption of azithromycin increased significantly from a DID of 2.59 in 2019 to 3.38 in 2020 and 6.09 in 2021. The increase was only observed in oral, and not in intravenous usage, suggesting self-administration of antibiotics. The observation can be explained by the fact that 2020 is marked by an increase in COVID-19 cases, and azithromycin was used indiscriminately as an immune modulator during COVID-19 infection [35–37]. Moreover, the increase in usage of fluoroquinolones and cephalosporins in 2021 can be further explained by the third wave of COVID-19 infections that were caused by the ß-variant of the severe acute respiratory syndrome coronavirus 2 (SARS-CoV-2) virus. COVID infections during the first wave, which started in March of 2020, remained self-limiting and required little to no medical intervention, in particular hospitalizations. The observed increase in consumption trends in the year 2021, overlays well with the third wave of infection which was caused by the ß-variant of the SARS-CoV-2 virus [38]. The wave started in March of 2021 and the country witnessed a surge in number of cases, where 4500 cases were reported in 24 hours, the largest number of positive cases that the country had witnessed since the start of the pandemic in Pakistan [39]. Additionally, the highest hospitalization rates together with significantly high ventilator occupancies were reported during the same wave, making it the deadliest wave of the pandemic in Pakistan. It is therefore likely that there was an increase in consumption of both intravenous and per-oral antimicrobials to prevent upper and lower-respiratory tract bacterial infections and to prevent nosocomial infections in hospitalized patients.

Previously, trends analysis of antimicrobial usage between 2014 and 2018 also reported increased usage of carbapenems and quinolones. Previous studies have indicated the non-judicious use of quinolones in hospitalized individuals, where a significant proportion of these antimicrobials are administered prophylactically to prevent hospital-acquired infections. Second, ciprofloxacin is widely used for the treatment of urinary tract infections, the most common complaint in adults requiring antibiotic treatment [40]. Additionally, quinolones are also prescribed extensively for the treatment of upper and lower respiratory tract infections, in most cases without sensitivity testing [33]. The use of third-generation cephalosporins (in particular cefoperazone + sulbactum) showed a net increase of 4.35 DID, where the increase was observed mostly in intravenous administration. Peroral usage of macrolides (clarithromycin) also showed a significant net increase of 1.3 DID during the study period. Similar to quinolones, macrolides are also used as broad-spectrum antimicrobials. In adults in particular, clarithromycin is prescribed for *Helicobacter pylori* infections, whereas in children, the most common usage is in the treatment of upper respiratory tract infections. Otitis infections and helicobacter infections are frequent in the Pakistani population, which may have led to the increased usage of these antimicrobials [41]. Noteworthy is the increased consumption of anti-MRSA antimicrobials, in particular linezolid, which belongs to the reserve class of antimicrobials [36, 38].

When stratified by regions, we observed a significantly high consumption of antimicrobials for all 3 years in Region 7 (Peshawar), Region 1 (Karachi), and Region 6 (Rawalpindi), whereas the least consumption was observed in Region 3 (Multan), Region 2 (interior Sindh), and Region 5 (Faisalabad). These observations suggest that consumption is higher in more urbanized communities and most likely is reflective of self-medication. Additionally, some of the largest tertiary care facilities associated with these regions are also contributing to the high consumption of antimicrobials. The most frequently used classes of drugs in these areas include quinolones, penicillins, and third-generation cephalosporins and macrolides. When compared to other regions, consumption of third-generation cephalosporin was high in Region 2 (interior Sindh). Another important observation made regionally is the increased use of intravenously administered antimicrobials in particular in Region 5 (Faisalabad) and Region 4 (Lahore). This can be correlated with wave 3 of COVID-19 infection. Punjab—in particular, larger cities in Punjab including Multan, Faisalabad and Lahore—remained the epicenter of the pandemic for Pakistan, where the highest number of COVID cases along with COVID-19 deaths were reported throughout the pandemic. The increase in the burden of viral infection might have led to the increase in the use of antimicrobials to prevent bacterial pneumonia or to treat ventilator-acquired, or hospital-acquired pneumonia and other infections [25, 29].

## CONCLUSIONS

Appropriate and judicious use of antimicrobials is an important public health priority. The current situation of antimicrobial usage in Pakistan is concerning and requires appropriate stewardship and drug review programs to be implemented in hospitals to prevent inappropriate prescription of antimicrobials. Development and implementation of guidelines are therefore necessary to restrict the use of antimicrobials for viral infections of the upper respiratory tract. For bacterial infections, antimicrobials should be prescribed after performing culture sensitivity on the isolates where appropriate. Strict sale regulations are required to limit self-medication.

### Study Limitations

The present study estimates the antimicrobial usage pre-COVID and post-COVID; however, the estimates are not correlated with rates of COVID infections in each region. Moreover, the study does not provide any data on use of antimicrobials after the pandemic diminished. It is likely that the usage might have changed post-pandemic, and analysis of these trends will provide more accurate estimates of usage.

## Notes

***Disclaimer.*** The views expressed here are those of the authors and not necessarily those of the UK Department of Health and Social Care or its management agent, Mott MacDonald; and, International Vaccine Institute.

***Financial support*.** This research was supported by Department of Health and Social Care, The UK through Fleming Fund country grant for Pakistan.

***Supplement sponsorship*.** This article appears as part of the supplement “Capturing Data on Antimicrobial Resistance Patterns and Trends in Use in Regions of Asia (CAPTURA),” funded by the Department of Health and Social Care's Fleming Fund using UK aid.
